# Examining cultural adaptations of the savvy caregiver program for Korean American caregivers using the framework for reporting adaptations and modifications-enhanced (FRAME)

**DOI:** 10.1186/s12877-024-04715-w

**Published:** 2024-01-20

**Authors:** Yuri Jang, Kenneth Hepburn, William E. Haley, Juyoung Park, Nan Sook Park, Linda K. Ko, Miyong T. Kim

**Affiliations:** 1 Edward R. Roybal Institute on Aging, Suzanne Dworak-Peck School of Social Work, University of Southern California, Los Angeles, USA; 2 Department of Social Welfare, Ewha Womans University, Seoul, South Korea; 3 Nell Hodgson Woodruff School of Nursing, Emory University, Atlanta, USA; 4 School of Aging Studies, University of South Florida, Tampa, USA; 5 School of Social Work, University of South Florida, Tampa, USA; 6 Department of Health Systems and Population Health, University of Washington, Seattle, USA; 7 School of Nursing, University of Texas at Austin, Austin, USA

**Keywords:** Evidence-based intervention, Cultural adaptation, FRAME, Dementia caregivers, Korean americans

## Abstract

**Background:**

The Framework for Reporting Adaptations and Modifications–Enhanced (FRAME) is a tool that systematically guides decision-making and reporting of adaptations made to evidence-based interventions. Using FRAME, we documented the process and outcomes of adapting the Savvy Caregiver Program (SCP) for Korean American dementia caregivers.

**Methods:**

Sequential adaptation was initiated with linguistic attunement, followed by pilot implementation and full adaptation. Our data-driven adaptation with multiple data sources and a feedback loop among multiple stakeholders yielded a total of 32 modifications, and each was coded according to the eight domains of FRAME: (1) what was modified, (2) who participated in recommending and deciding the modification to be made, (3) when the modification occurred, (4) whether the modification was planned, (5) whether the modification was fidelity-consistent, (6) whether the modification was temporary, (7) at what level of delivery, the modification was made, and (8) why the modification was made.

**Results:**

The areas of adaptation were evenly distributed across context (37.5%), content (31.2%), and training (31.2%). The primary reasons for modification were for engagement (62.5%), followed by fit with recipients (43.8%) and outcome improvement (31.1%). About 66% of the modifications were applied to the entire target group, and all modifications were fidelity-consistent.

**Conclusions:**

The FRAME categorization provided a detailed understanding of the process and nature of adapting the SCP and served as a foundation for further implementation and scale-up. FRAME not only serves as a guide for adapting evidence-based interventions but also promotes their replicability and scalability.

## Background

When an evidence-based intervention (EBI) is implemented in a new setting, for a new population, and/or with a new mode of delivery, change is inevitable. Despite the fact that the implementation of EBIs within real-world settings is a dynamic process [1] and that it is important to balance fidelity with fit when an EBI is adapted for a new context [2–4], researchers have lacked a tool to systematically guide their decision making in adapting EBIs and inform their reports of adaptations [5]. To broaden the reach and impact of EBIs yet maintain their integrity, such tools are indispensable [1–5].

The Framework for Reporting Adaptations and Modifications–Enhanced (FRAME) [6, 7] constitutes a response to the need for such a tool. FRAME comprises eight domains: (1) what is modified (i.e., areas/aspects of the modification), (2) who participates in recommending the modification and deciding about it (i.e., who drives the change), (3) when the modification is made, (4) whether the modification was planned or in response to the needs emerged during implementation, (5) whether the modification is consistent with the intention of the EBI (i.e., fidelity), (6) whether the modification represents a temporary drift or a permanent change that applies to the entire program, (7) at what level of delivery, the modification was made (i.e., for whom the modification is made), and (8) why the modification was made (i.e., reasons, goals, and rationales). Table 1 summarizes the FRAME domains, including descriptions and examples. FRAME has been applied in adapting an EBI in a new setting (e.g., Montessori-based activity programming in nursing homes) [8], for a new population (e.g., the Adult Day Services Plus intervention for Hispanic/Latino dementia caregivers) [9], and for a new delivery mode (e.g., skills training in affective and interpersonal regulation for posttraumatic stress disorder for peer delivery) [10]. The use of FRAME improves the efficiency of adaptations, enables their standardization, and facilitates empirical evaluations of an adapted intervention’s outcomes [9, 10].

**Table 1:** FRAME domains

Domain	Description	Examples
1. What	Areas/aspects modified	• Content • Context • Training • Evaluation • Implementation
2. Who	Individuals who participated in recommending and deciding the modification to be made (i.e., the driver of change)	• Researcher • Intervention developer • Participant • Interventionist/service provider • Administrator • Policy maker
3. When	Timing of the adaptation	• Pre-implementation • Pilot-implementation • Implementation • Scale-up
4. Planning	Whether the modification was planned	• Planned (proactive adaptation) • Unplanned (reactive adaptation)
5. Fidelity	Whether the modification was in line with the core element of the program	• Fidelity-consistent • Fidelity-inconsistent
6. Temporality	Whether the modification was a temporary drift	• Transient change specific to a situation • Broad change for the extended period
7. Delivery level	At what level of delivery, the modification was made (i.e., for whom the modification was made)	• Entire target group • Specific target subgroup • Entire practitioner group • Specific practitioner subgroup • Entire organization • Specific part of the organization
8. Why	Reasons for the modification	• To improve fit with recipients • To increase reach/engagement • To improve effectiveness/outcomes • To reduce cost

*Note*. FRAME = Framework for Reporting Adaptations and Modifications–Enhanced

The Savvy Caregiver Program (SCP) is an evidence-based psychoeducational program, delivered by Savvy-certified trainers to groups of dementia caregivers either in person or online [11, 12]. The course content of the 6-week program includes introduction to dementia, caregiver self-care, the effect of dementia on the performance of everyday tasks and activities, contented involvement (i.e., helping caregivers remain calm and pleasant while interacting with the care recipient), a decision-making guide for family caregivers, and strengthening family caregiving arrangements. The program targets enhancement of caregiver self-efficacy (i.e., confidence in using specific skills addressed through caregiver training) as the mechanism of action that promotes caregiver outcomes [11, 12]. Through a combination of instruction, modeling, active learning and practice, and coaching, the program seeks to strengthen caregivers’ knowledge and understanding of the progressive consequences of dementia on individuals under their care. As a transportable training program offered in community settings, SCP has helped to improve caregiving outcomes and caregivers’ well-being in diverse communities [12]. Efforts have been made to adapt SCP for Spanish-speaking caregivers in consideration of language and family-oriented cultures [13]. However, no systematic approaches to cultural adaptation and the process documentation have been made [9], and the program has rarely reached ethnic minorities who speak languages other than English or Spanish [14].

We therefore adapted the SCP linguistically and culturally for Korean American dementia caregivers, branded as K-Savvy (6-week online psychoeducation delivered in Korean). In the pilot implementation, K-Savvy was shown to be not only feasible and acceptable [15] but also efficacious in reducing depressive symptoms of dementia caregivers [16]. In efforts to establish a systematic foundation for further implementation and scale-up, we recognized the necessity of documenting the process and outcomes of our cultural adaptations. Here, we review the modifications that we made in adapting the SCP as K-Savvy as a means of both describing this intervention modification in great detail and illustrating the value of FRAME for future intervention projects.

## Methods

### Overview

Because cultural responsiveness requires more than simple translation [2–4, 9], adaptation of the SCP proceeded sequentially. First, we translated the SCP caregiver manual into Korean and certified two lay individuals who were bilingual in English and Korean as Savvy trainers. Next, we pilot tested the program. During the six-week period in February and March, 2022, the two trainers delivered the translated SCP program online in Korean to groups of six to seven caregiver participants, who were recruited through multiple sources, including a previously established network of dementia caregivers, public advertisement, and referrals from social service providers. Participants were self-identified Korean Americans residing in the greater Los Angeles area who were caregivers of a family member with dementia, spoke English less than *very well*, and had access to computer and the Internet. Participants had a low to moderate level of depressive symptoms (the Patient Health Queationnaire-9 [17] score < 20) and no prior exposure to the SCP.

Data from the translation, certification, and pilot testing (e.g., session observations, session video recordings, quantitative and qualitative assessments with caregiver participants and trainers, field notes and meeting logs, trainer fidelity checklists) then informed our subsequent adaptation. The process involved the collaboration of multiple stakeholders: caregiver participants (*n* = 13), trainers (*n* = 2), the intervention developer (*n* = 1), other research team members (*n* = 4), and members of an advisory panel (*n* = 11). The advisory panel included five social service providers in Korean communities and six University-affiliated researchers in the field of aging, culture, immigration, and health. From the data, our research team generated a list of recommended changes for full cultural adaptation, which was reviewed by the SCP’s developer for fidelity and by the advisory panel for cultural and contextual relevance. Using a feedback loop and strategies to balance fidelity and fit [2–4], we made several modifications to address logistical, technical, and cultural issues in fully adapting the SCP for K-Savvy [15].

### Coding of adaptations using FRAME

Two research team members who were involved in the pre-implementation and pilot-testing phases developed an initial chart, listing all modifications and coding each modification according to the FRAME domains. The chart was reviewed by the SCP developer and other research team members. Discrepancies in coding were discussed to reach consensus. The finalized FRAME chart was then evaluated to demonstrate the frequency and percentage of each category and characterize the nature of the adaptations made.

## Results

In creating K-Savvy, a total of 32 modifications were made to the SCP. Although each of these modifications are detailed elsewhere [15], in the present study our aim was to document them using the FRAME categorization. Table 2 shows how these modifications were categorized within the FRAME domains, and summary statistics are provided in Table 3. The areas of adaptation were evenly distributed across context (37.5%), content (31.2%), and training (31.2%). The research team made recommendations and decisions for all modifications; others who contributed to changes included trainers (62.5%), caregiver participants (34.3%), advisory panel members (34.3%), and the SCP developer (25%). Since multiple stakeholders were involved in these recommendations and changes, totals are shown as greater than 100%. Input from the SCP developer was critical for maintaining fidelity to the SCP. For example, given the length of the caregiver manual (290 pages), a shorter version was suggested. However, this was rejected by the SCP developer in order to preserve all essential elements of the original SCP manual. Instead, weekly handouts outlining the manual’s content were provided as a reference. Augmenting content to reframe the SCP’s individualistic values (e.g., personhood, self-care, self-efficacy) for the target group’s collectivistic cultural context was strongly endorsed by the SCP developer, resulting in the modification of content. Apart from the translation of the manual and the certification of the bilingual trainers, most of the modifications (78.1%) were made after pilot implementation. Five modifications to the SCP (15.6%) were planned during pre-implementation and reconfirmed during pilot implementation. For example, the use of the word “Savvy” without translation into Korean was planned before the pilot test, and this was strongly endorsed by both caregiver participants and trainers. In qualitative interviews conducted after the pilot test, participants and trainers reported that the word Savvy fit well with the program and was impactful. They also suggested branding the program as K-Savvy and including a brief discussion of the program’s name during the first session, which the research team and the SCP developer approved.

**Table 2:** Adaptations for K-Savvy using FRAME

	Adaptations Made	What	Who	When	Planned	Fidelity-consistent	Temporary drift	At what level of delivery	Why
1	Translating SCP manuals into Korean	Content	RT	Pre	Proactive	Yes	No	Entire target group	Fit
2	Training Korean-speaking trainers	Context	RT, ID	Pre	Proactive	Yes	No	Entire target group	Fit
3	Keeping the English word “Savvy” without translation	Content	P, T, RT, ID	Pre/Pilot	Proactive/ Reactive	Yes	No	Entire target group	Fit
4	Including a brief discussion of the program’s name during the first session	Content	T, RT, ID	Pilot	Reactive	Yes	No	Entire target group	Fit/ Engagement
5	Branding the program as “K-Savvy”	Context	RT, AP, ID	Pilot	Reactive	Yes	No	Entire target group	Fit/ Engagement
6	Keeping the 6-week frequency	Context	P, T, RT	Pre/Pilot	Proactive/ Reactive	Yes	No	Entire target group	Engagement
7	Increasing the session length to 90 min	Context	P, T, RT	Pilot	Reactive	Yes	No	Entire target group	Engagement/ Outcome
8	Limiting the number of participants to six or seven	Context	P, T, RT	Pre/Pilot	Proactive/ Reactive	Yes	No	Entire target group	Engagement/ Outcome
9	Considering participants’ characteristics in class assignment	Context	T, RT	Pilot	Reactive	Yes	No	Entire target group	Engagement/ Outcome
10	Preparing trainers to better attend to diversity among class participants	Training	RT, AP	Pilot	Reactive	Yes	No	Entire trainers	Engagement/ Outcome
11	Allowing participants to attend an alternative class in case of schedule conflict	Context	P, T, RT	Pilot	Reactive	Yes	Yes	Specific target subgroup	Engagement
12	Providing weekly-handouts to outline the content in the manual	Context	P, T, RT, ID	Pilot	Reactive	Yes	No	Entire target group	Engagement
13	Using an introductory video clip with Dr. Hepburn’s endorsement of Savvy trainers	Context	RT, AP	Pilot	Reactive	Yes	No	Entire target group	Engagement
14	Enhancing the pre-session prep/post-session debriefing sessions for trainers	Training	T, RT	Pre/Pilot	Proactive/ Reactive	Yes	Yes	Entire trainers	Outcome
15	Offering a booster training session for trainers with additional training needs	Training	T, RT	Pilot	Reactive	Yes	Yes	Specific trainer subgroup	Outcome
16	Improving in-session time allocation for discussion	Training	P, T, RT	Pilot	Reactive	Yes	No	Entire trainers	Engagement
17	Offering a Savvy Caregiver Certificate for those who complete the program	Context	RT, AP	Pilot	Reactive	Yes	No	Entire target group	Engagement
18	Utilizing the pre-meeting with participants to help them become technically prepared	Context	P, T, RT	Pre/Pilot	Proactive/ Reactive	Yes	No	Entire target group	Outcome
19	Enhancing technical training for trainers	Training	T, RT	Pilot	Reactive	Yes	No	Entire trainers	Outcome
20	Augmenting course contents to reframe the seemingly individualistic SCP values in the context of collectivistic cultures	Content	RT, AP, ID	Pilot	Reactive	Yes	No	Entire target group	Fit/ Engagement/ Outcome
21	Addressing participants’ unique situations and foster everyone’s sense of belonging	Training	RT, AP	Pilot	Reactive	Yes	No	Entire trainers	Fit/ Engagement
22	Enhancing coverage on caregiving resource maps in Session 5 and caregiving arrangement types in Session 6	Content	RT, ID	Pilot	Reactive	Yes	No	Entire target group	Fit
23	Including how to handle sensitive topics in trainer training	Training	T, RT	Pilot	Reactive	Yes	No	Entire trainers	Engagement
24	Developing a list of potential questions and responses for trainers	Training	RT, AP	Pilot	Reactive	Yes	No	Entire trainers	Engagement
25	Training trainers on how to facilitate discussions, make all participants engaged	Training	T, RT	Pilot	Reactive	Yes	No	Entire trainers	Engagement
26	Including a brief talk on group discussion encouraging all participants’ active engagement in the first session	Content	T, RT	Pilot	Reactive	Yes	No	Entire target group	Engagement
27	Setting rules and expectation for class participation addressing traditional culture influenced communication style	Context	T, RT	Pilot	Reactive	Yes	No	Entire target group	Fit/ Engagement
28	Including coaching strategies for positive reinforcement in trainer training	Training	RT, AP	Pilot	Reactive	Yes	No	Entire trainers	Outcome
29	Including Korean subtitles in the video clips	Content	P, T, RT, AP	Pilot	Reactive	Yes	No	Entire target group	Fit
30	Replacing some photos in the manual with those featuring Korean/Asian individuals	Content	P, T, RT, AP	Pilot	Reactive	Yes	No	Entire target group	Fit
31	Using culturally relevant examples	Content	P, T, RT	Pilot	Reactive	Yes	No	Entire target group	Fit
32	Incorporating culturally specific discussions in each session	Content	RT, AP, ID	Pilot	Reactive	Yes	No	Entire target group	Fit/ Engagement

*Note*. P = participant, T = trainer, RT = research team, AP = advisory panel, ID = intervention developer

**Table 3:** FRAME summary of adaptations for K-Savvy (*N* = 32)

	n (%)
What was modified	
Context	12 (37.5%)
Content	10 (31.2%)
Training	10 (31.2%)
Who participated in recommending and deciding the modification	
Participant	11 (34.3%)
Trainer	20 (62.5%)
Research team	32 (100%)
Advisory panel	11 (34.3%)
Intervention developer	8 (25.0%)
When the modification occurred	
Pre-implementation phase only	2 (6.2%)
Pilot implementation phase only	25 (78.1%)
Both phases	5 (15.6%)
Whether the modification was planned	
Proactive change only	2 (6.2%)
Reactive change only	25 (78.1%)
Both proactive and reactive change	5 (15.6%)
Whether the modification was fidelity-consistent	
Consistent	32 (100%)
Inconsistent	0 (0%)
Whether the modification was a temporary drift	
Yes	3 (9.4%)
No	29 (90.6%)
At what level of delivery, the modification was made	
Entire target group	21 (65.6%)
Specific target subgroup	1 (3.1%)
Entire trainer group	9 (28.1%)
Specific trainer subgroup	1 (3.1%)
Why the modification was made (i.e., reasons, goals, rationales)	
To improve fit with recipients	14 (43.8%)
To improve engagement	20 (62.5%)
To improve outcome	10 (31.1%)

*Note*. In the domains of Who and Why, multiple entries were allowed, so percentages add up to more than 100%

Planning was closely aligned with timing. All modifications made during pre-implementation were planned and proactive, whereas those made after pilot implementation were unplanned and reactive because they were in response to the needs emerged during implementation. The five modifications that spanned both phases were both proactive and reactive. Given the study design with data-driven adaptation and close alignment with the SCP [15], most modifications (94%) were either fully or partially reactive, and all ensured fidelity. Only three modifications represented temporary drifts specific to pilot implementation, whereas the rest permanently applied to the entire program. Attending an alternative class was the only modification applicable to a specific target subgroup with a schedule conflict during the pilot implementation. Over a quarter of the modifications (28.1%) applied to the trainer group, and only one modification applied to a specific trainer subgroup. Booster training sessions were suggested only for trainers who needed additional training. The primary reasons for modification were for engagement (62.5%), fit with recipients (43.8%), and outcomes (31.1%). Many efforts to improve fit with recipients by addressing their linguistic and cultural needs were also linked with the goals of enhancing participants’ engagement and intervention outcomes.

## Discussion

In this study, we documented the process and outcomes of adapting the Savvy Caregiver Program (SCP) for Korean American dementia caregivers using FRAME. In implementing an evidence-based intervention (EBI) in a real-world setting, it is necessary to make changes to fit the culture, context, and characteristics of the target group [1–5]. Cultural adaptation is an important step that broadens the reach and impact of EBIs and potentially reduces inequities in care [2–4]. In dementia caregiving, interest in tailoring caregiver interventions for particular cultural or ethnic groups continues to grow [18–20]; however, there has been lack of systematic approaches [9]. FRAME has emerged as a useful tool that can organize and document the process and outcomes of cultural adaptation.

In our cultural adaptation of the SCP as K-Savvy for Korean American dementia caregivers, a total of 32 modifications were made and each of them was assessed with the use of the eight FRAME domains. FRAME allowed us to systematically document and evaluate modifications of the SCP in terms of adaptation areas, responsible parties, timing, reasons, and other characteristics pertaining to planning, fidelity, temporality, and delivery level. This categorization reflected the early stage of K-Savvy development, in which the program was designed for intervention fidelity and fit for our target population. Data-driven assessment based on feedback from caregiver participants and trainers identified the needs and areas for modifications, and input from community and research partners helped us determine the contextual and cultural relevance of the suggested changes. Furthermore, the SCP developer played a key role in the process by ensuring that the integrity of the program was not compromised by the modifications. The FRAME domains informed our identification of the target population’s needs and how they could be addressed, and these domains provided a detailed understanding of the process and nature of adapting the SCP. It should be noted that this study is a small pilot project in which we assessed implementation on the level of participants and trainers. In the future, we will scale up implementation to the organizational and system level, which may entail broader reasons for modifications (e.g., to reduce cost, to accommodate organizational needs).

## Conclusions

Integrating cultural adaptation and implementation science [9, 20, 21], FRAME is an efficient guide for EBI adaptation, and it promotes replicability and scalability of adapted interventions. The broad application of FRAME is highly recommended for intervention adaptations, since it can allow for more detailed description of such adaptations and more consistent approaches to this key step for interventions. Given the increasing diversity in our society, the systematic use of an adaptation tool will facilitate the harmonization and documentation effort across diverse populations.

## Data Availability

The dataset used in the current study is available from the corresponding author upon request.
